# POCT errors can lead to false potassium results

**DOI:** 10.1515/almed-2021-0079

**Published:** 2021-12-30

**Authors:** Antonio Buño, Paloma Oliver

**Affiliations:** Clinical Laboratory Department, Hospital Universitario La Paz, Madrid, Spain

**Keywords:** blood gases analysis, hemolysis, point-of-care-testing (POCT), pseudohyperkalemia

## Abstract

Point-of-care-testing (POCT) facilitates rapid availability of results that allows prompt clinical decision making. These results must be reliable and the whole process must not compromise its quality. Blood gas analyzers are one of the most used methods for POCT tests in Emergency Departments (ED) and in critical patients. Whole blood is the preferred sample, and we must be aware that hemolysis can occur. These devices cannot detect the presence of hemolysis in the sample, and because of the characteristics of the sample, we cannot visually detect it either. Hemolysis can alter the result of different parameters, including potassium with abnormal high results or masking low levels (hypokalemia) when reporting normal concentrations. Severe hyperkalemia is associated with the risk of potentially fatal cardiac arrhythmia and demands emergency clinical intervention. Hemolysis can be considered the most frequent cause of pseudohyperkalemia (spurious hyperkalemia) or pseudonormokalemia and can be accompanied by a wrong diagnosis and an ensuing inappropriate clinical decision making. A complete review of the potential causes of falsely elevated potassium concentrations in blood is presented in this article. POCT programs properly led and organized by the clinical laboratory can help to prevent errors and their impact on patient care.

## Introduction

Based on Price’s and Hicks’ definition point-of-care-testing (POCT) are those assays of blood and other body fluids or examinations of suspensions and smears of cells, crystals, not undertaken in central or satellite clinical laboratories, carried out close to the tested patient with the assumption that test results will be available instantly or in a very short timeframe so that their results can quickly lead to changes in patient care [1].

The rapid availability of results that allows prompt clinical decision-making is the main reason for using POCT and avoids the need to send specimens to a central laboratory facility. Results for POCT need to be reliable to contribute to better patient care. The healthcare professional running these tests does not need to be qualified in laboratory disciplines, but they must be adequately trained and certified by laboratory professional that must supervise this critical part of the POCT process. Despite its potential benefits such as reduced turnaround time (TAT) or fewer preanalytical errors due to the simplification of processes, few studies have assessed its impact. POCT devices have become increasingly available, in terms of the repertoire of tests, number of suppliers, and availability of methods and devices. Utilization is expected to increase further [2].

The World Health Organization defines medical error as an adverse event or near miss that is preventable with the current state of medical knowledge. An adverse device event is any incident in which the use of medical equipment may have resulted in an adverse outcome for the patient. And a preventable adverse event is an adverse event that would not have occurred if the patient had received ordinary standards of care appropriate for the time [3]. When considering medical errors in clinical laboratory testing, are mostly failures of planned testing actions. These also include POCT [4]. In a study of stat test errors published by Plebani et al., of 189 clinician-discovered errors, they found that three quarters of them (140 [70%]) did not affect patient’s outcome, but that about a fifth (37 [19.6%]) were associated with inappropriate investigations resulting in an unjustifiable increase in costs, and that one in 16 (12 [6.3%]) led to inappropriate care or inappropriate modification of therapy [5].

We can divide POCT errors into the three phases of the process, preanalytic, analytic, and postanalytic. Most of them have been recognized in the preanalytic phase as occurs with errors in the stat laboratory where some authors found that the distribution of mistakes was: preanalytical 68.2%, analytical 13.3%, and postanalytical 18.5% [5]. Different steps of the preanalytic phase can be potential areas for errors like test ordering, patient and specimen identification, specimen collection that include inappropriate or inconsistent specimen type, volume, or application to the POCT device’s testing surface or reaction chamber and the last one, specimen evaluation or assessment of specimen attributes. Kost distinguished between two types of attributes that may influence whole blood assays, patient-related like anemia or leukocytosis and those which are collection related, mostly hemolysis and clotting [6].

Regarding the analytical phase, the first step with an opportunity for error may be due to method calibration. The next step for defects may be specimen/reagent interaction either because of patient-related native interferences, specimen-related nontarget influences or the existence of potential matrix effects. Generation, interpretation, and validation of results are the next steps at this phase with the potential to cause errors [4]. Nevertheless, the emerging consensus is that in the POCT process, error reduction through advances in technology can almost eliminate “analytical” errors. So, today’s emphasis must shift to the entire POCT process [7, 8].

In the final phase of the POCT process (postanalytical), defects can occur at steps of report formatting, critical value reporting, other result reporting, and lastly, the ultimate step of report management that includes verification, preservation, storage, and retrieval [4].

Monitoring of quality indicators in all these phases is a very valuable tool in reducing errors in POCT [8, 9].

The US Institute of Medicine recognizes two kinds of medical POCT-related errors that impact patient safety: initiating the incorrect therapeutic action and/or failing to recognize the significance of the test result, and failure to take the necessary action [10]. This is another way of considering errors in POCT.

Blood gas analyzers are one of the most used methods for POCT tests in Emergency Departments (ED) or hospitalized patients. Many of these analyzers can also measure other parameters such as potassium and glucose and lactate among others. Whole blood is the preferred sample used in these analyzers. But we must be aware that when using this sample hemolysis can occur particularly associated with the collection of blood step specially in those clinical scenarios with a high stress environment such as the EDs and Critical Care Units [11]. *In vitro* hemolysis typically occurs during the preanalytical phase of testing particularly during the collection process itself or the transportation of the sample to the analyzer. Some other factors can heavily influence the appearance of hemolysis. For example, blood collected through venous catheters has been described to have an increased risk of hemolysis compared to blood collected using butterflies or straight needles [12].

We also need to consider that unlike chemistry analyzers in central laboratories that can routinely quantify the concentration of free hemoglobin in plasma and serum samples and report a so-called hemolysis index, blood gases analyzers cannot detect the presence of hemolysis in the sample, and because of the characteristics of the sample we cannot visually detect it either. Other strategies have been utilized using machine learning algorithms with the potential to detect laboratory errors based on the recognition of patterns in commonly requested multianalyte panels and some authors proposed a predictive analytics model to flag suspiciously elevated potassium results [13]. Hemolysis can alter the result of different parameters, including potassium with abnormal high results or masking normal or very low levels (hypokalemia) when reporting normal or low concentrations. So, recognition of hemolysis when using POCT methods can be really challenging.

## Hyperkalemia and hemolysis

Hyperkalemia is conventionally defined as a serum or plasma potassium concentration >5.5 mmol/L or >5.0 mmol/L, respectively [14]. Severe hyperkalemia, usually defined as serum or plasma potassium greater than 6.5–7.0 mmol/L is associated with risk of potentially fatal cardiac arrhythmia and warrants emergency clinical intervention [14]. The prevalence of this electrolyte disturbance was found to be as high as 10% in hospitalized patients [6]. Hyperkalemia is often asymptomatic and discovered on routine laboratory tests. When symptoms are present, they are non-specific. Disorders of plasma potassium can have profound effects on the nerve, muscle, and cardiac function and it may be associated with severe complications such as fasciculation, paresthesia, and arrhythmias (severe hyperkalemia can cause global paralysis). Hyperkalemia may produce progressive characteristic abnormalities on the electrocardiogram (ECG), including peaked T waves, flattening or absence of P waves, widening of QRS complexes, and sine waves [15].

Evaluation of a patient with hyperkalemia should include a review of the medical history to identify potential contributing factors. Potential causes like renal failure, diabetes, adrenal insufficiency, and the use of drugs that cause hyperkalemia must be assessed. Laboratory blood and urine tests should be directed toward causes and may be useful in distinguishing between renal and non-renal causes of hyperkalemia [16].

We can divide its causes into biological like renal failure, increased potassium intake, or administration of certain drugs such as β-blockers, digoxin, and potassium sparing diuretics, among others and preanalytical problems such as blood sample hemolysis, prolonged tourniquet placement, fist clenching, sample contamination from infusive routes, prolonged storage of uncentrifuged blood, as well as by leukocytosis or thrombocytosis [14, 17, 18].

Hemolysis is defined as the rupture of erythrocytes resulting in the release of its intracellular components in plasma or serum. Hemolysis may occur either *in vitro* or *in vivo*, with *in vivo* hemolysis accounting for <2% of cases [19]. We can define *in vitro* hemolysis as the breakdown of erythrocytes occurring during collection, management, transportation, and storage of biological samples [20]. It is a substantial problem in laboratory diagnostics because results obtained on spuriously hemolyzed specimens are unreliable. Ninety-eight percent of the body’s potassium is in the intracellular fluid (concentration about 140 mmol/L), with only 2% in the extracellular fluid (3.8–5.0 mmol/L) [21]. This explains why a small percentage of hemolysis can have a big impact on serum or plasma potassium concentrations. Hemolysis can be considered the most frequent cause of pseudohyperkalemia (spurious hyperkalemia) or pseudonormokalemia when a true hypokalemia exists masking low levels and may hence be accompanied by a wrong diagnosis of hyperkalemia and an ensuing inappropriate clinical decision making [22]. Some authors found that the frequency of occult hemolysis is significant in whole blood samples referred to the laboratory for blood gas analysis, and typically comprised between 4 and 13% [21, 22]. Table 1 lists common causes of pseudohyperkalemia (spurious hyperkalemia) or pseudonormokalemia (spurious normokalemia). From a conceptual point of view, we can classify these causes into four groups: 1) those related to the preanalytical phase of the POCT process specially those associated with the collection and handling of samples; 2) the analytical phase; 3) conditions related to the patient itself; 4) and causes that have to do with organizational circumstances [23]. The largest percentage of errors occurs in the preanalytical phase particularly during the collection of the sample [24].

**Table 1:** Potential causes of pseudohyperkalemia or pseudonormokalemia in POCT.

A.	Related to the preanalytical process
	– An excessive tourniquet
	– Repeated fist clenching
	– Inappropriate needle size (narrow gauge)
	– Use of syringe and needle rather than vacuum tube collection systems
	– Non-standard (i.e., other than antecubital fossa) venipuncture site
	– Excess blood flow rate (vacuum)
	– EDTA contamination (via syringe needle contamination or incorrect order of draw)
	– Traumatic venipuncture (squeezing the puncture site)
	– Blood collected from a vein into which potassium is infused
	– Overly vigorous sample mixing
	– Prolonged storage of uncentrifuged blood
	– Delayed analysis (ongoing *in vitro* glycolysis, leakage of potassium)
	– Blood gas syringe transported in direct contract with ice
B.	Related to the analytical process
	– Laboratory error (analytical)
	– Excessive injection pressure while introducing the sample in the analyzer
C.	Related to the patient
	– Severe leukocytosis (>150 × 10^9^/L)
	– Marked increase in platelet count (thrombocytosis) (>500 × 10^9^/L)
	– Inherited defects in erythrocyte membrane structure (brittle RBC): familial pseudohyperkalemia and hereditary stomatocytosis
	– Elderly patients or newborns
D.	Related to organizational issues
	– Increased workload and stressed environment
	– Insufficiently trained operators

POCT, point-of-care-testing.

In patients without a predisposition to hyperkalemia, serum potassium to rule out spurious hyperkalemia may be needed, unless ECG changes suggest that emergency treatment is necessary. Nevertheless, we must be aware that ECG changes correlate poorly with the degree of potassium disturbance [14].

The prevalence of unsuitable specimens referred for blood gas analysis ranges between 1.2 and 3.7% [24]. Several parameters have been demonstrated to be affected by spurious hemolysis. Some of them are clearly described in the literature like the substantial increase in the concentration of potassium due to the different gradient of concentration between the blood cell and the plasma or the decrease observed in the concentration of ionized calcium, but some others can also be affected like pO2, pCO2, sO2, and COHb. Pathophysiological mechanisms underlying these variations of blood gases should hence be attributable to complex and unidentified causes related to the presence of cell-free hemoglobin, as well as to the release of a variety of intracellular molecules [20]. It would be desirable that manufacturers of blood gas analyzers could develop new instrumentation capable of identifying the presence of interfering substances in whole blood samples like hemolysis, lipemia, and icterus in whole blood specimens, as for clinical chemistry and coagulation testing in serum or plasma samples.

Wilson et al. published in 2018 an ambispective study where they compared hemolysis rates in blood gas samples taken in an intensive care unit (ICU) with those taken in an ED. They found that hemolysis was more frequent and of greater severity in samples from ED than in those from ICU and higher in whole blood than serum samples from both ICU and ED. Thirteen percent of ED potassium results and 4% of ICU potassium results obtained from whole blood samples by point-of-care analysis would have been considered as potentially inaccurate due to hemolysis (mild or severe) [25].

## POCT vs. central laboratory testing

Electrolyte abnormalities can trigger life-threatening events in emergencies. Hence, rapid assessment of electrolyte abnormalities plays a decisive role in this scenario for rapid therapeutic management. POCT considerably reduces TAT when used for the measurement of electrolytes in the ED and adult ICU when compared with central laboratory testing [26]. However, contradictory results were observed while analyzing the exact values of major electrolytes by either POCT or central laboratory testing methods [27], [28], [29]. Chako et al. compared whole blood electrolyte with POCT device vs. serum electrolyte at the central laboratory. They observed that the difference in values was large, particularly of potassium with values below 3 mmol/L. However, the difference for potassium values greater than 3 mmol/L was observed to be uniform and in good concordance [27]. The characteristics of electrodes used for analysis may also influence the observed differences. While the majority of the POCT analyzer uses direct ion-selective electrodes (measure the activity of ions in plasma), the central laboratory analyzers have indirect ion-selective electrodes that measure the activity of ions in a prediluted sample, being affected by dissolved solids such as triglycerides or proteins. Morimatsu et al. found similar results for sodium and chloride when comparing anion gap differences calculated using values from a point-of-care blood gas and electrolyte analyzer and the central laboratory automated blood biochemistry analyzer. The authors concluded that this might lead clinicians to different assessments of acid-base and electrolyte status [28].

As mentioned before, a clear disadvantage of POCT vs. central laboratory is the limited capacity to detect hemolysis in POCT devices when compared to conventional chemistry analyzers.

## What can we do?

When compared with central laboratory POCT can eliminate inefficiencies, although it may suffer from unique preanalytic, analytic, and postanalytic errors that result when human operators perform tests at the bedside. Preventing those errors and their impact on patient care will help to improve medical and economic outcomes. POCT programs properly led and organized by the clinical laboratory can help to achieve this. Strategies with secure operator identification that validate them before analysis and adequate performance with quality assurance plans and data transfer (connectivity) are also necessary [6].

Development, implementation, and validation of performance using meaningful and reliable key performance indicators (KPIs) is recommended to improve POCT quality. Its identification and selection will be driven by the vision, values, and goals of the organization. They should focus on processes where errors that can affect patient safety are more feasible and therefore should be selected based on risk across the total testing process. A multidisciplinary effort is required to achieve the desired performance goals and improve outcomes. The evaluation of KPIs over time could determine a set of quality indicators and the implementation of improvement actions with POCT led by laboratory medicine, to achieve safer and better patient care [30, 31].

Proper training of the personnel in charge of blood collection and being aware of the factors that may induce hemolysis as well as implementation of standardized collection procedures are essential to minimize hemolysis rates and improve the reliability of laboratory results.

Technological improvements in blood gas analyzers are also desirable. Advanced blood gas analyzers use to incorporate cooximeters capable to measure total hemoglobin in whole blood. Perhaps developments in the algorithms and methods used for this purpose could develop new instrumentation capable of identifying the presence of interfering substances in whole blood samples like hemolysis.

The correct training of the healthcare professional running these tests combined with further developments in new analyzers capable to detect hemolysis in the context of a properly organized POCT programs led and organized by the clinical laboratory will be the three cornerstones to help to detect and decrease hemolysis rates. The ability to minimize its incidence during the preanalytical phase of POCT would help to substantially mitigate the negative effects on patient management due to pseudohyperkalemia or pseudonormokalemia due to false potassium results at the point-of-care.
